# Next-Generation Bioinformatics Approaches and Resources for Coronavirus Vaccine Discovery and Development—A Perspective Review

**DOI:** 10.3390/vaccines9080812

**Published:** 2021-07-22

**Authors:** Rahul Chatterjee, Mrinmoy Ghosh, Susrita Sahoo, Santwana Padhi, Namrata Misra, Visakha Raina, Mrutyunjay Suar, Young-Ok Son

**Affiliations:** 1 School of Biotechnology, Kalinga Institute of Industrial Technology (KIIT), Bhubaneswar 751 024, India; rahulksbt@gmail.com (R.C.); ss.sahoo8976@gmail.com (S.S.); namrata@kiitincubator.in (N.M.); vraina@kiitbiotech.ac.in (V.R.); 2 KIIT-Technology Business Incubator (KIIT-TBI), Bhubaneswar 751 024, India; mringhs2010@gmail.com (M.G.); santwana@kiitincubator.in (S.P.); 3 Department of Animal Biotechnology, Faculty of Biotechnology, Jeju National University, Jeju 63243, Korea; 4 Bio-Health Materials Core-Facility Center, Jeju National University, Jeju 63243, Korea; 5 Practical Translational Research Center, Jeju National University, Jeju 63243, Korea; 6 Interdisciplinary Graduate Program in Advanced Convergence Technology and Science, Jeju National University, Jeju 63243, Korea

**Keywords:** bioinformatics resources, coronavirus, immunoinformatics, vaccine, epitope

## Abstract

COVID-19 is a contagious disease caused by severe acute respiratory syndrome coronavirus 2 (SARS-CoV-2). To fight this pandemic, which has caused a massive death toll around the globe, researchers are putting efforts into developing an effective vaccine against the pathogen. As genome sequencing projects for several coronavirus strains have been completed, a detailed investigation of the functions of the proteins and their 3D structures has gained increasing attention. These high throughput data are a valuable resource for accelerating the emerging field of immuno-informatics, which is primarily aimed toward the identification of potential antigenic epitopes in viral proteins that can be targeted for the development of a vaccine construct eliciting a high immune response. Bioinformatics platforms and various computational tools and databases are also essential for the identification of promising vaccine targets making the best use of genomic resources, for further experimental validation. The present review focuses on the various stages of the vaccine development process and the vaccines available for COVID-19. Additionally, recent advances in genomic platforms and publicly available bioinformatics resources in coronavirus vaccine discovery together with related immunoinformatics databases and advances in technology are discussed.

## 1. Introduction

Pandemics of crippling viral infections such as influenza, smallpox, Middle East respiratory syndrome-related coronavirus (MERS), severe acute respiratory coronavirus 1 syndrome (SARS-CoV-1), Ebola, and Zika have occurred many times in human history [1]. The extreme acute respiratory coronavirus 2 syndrome (SARS-CoV-2) recently emerged, and the World Health Organization (WHO) formally proclaimed it a pandemic on 11 March 2020. Since the first incidence of the disease was observed in the Chinese city of Wuhan, the entire world has been fighting to keep this outbreak under control. Promptly afterward, rising infection figures significantly inflated, resulting in a high number of fatalities in every corner of the globe.

The research on viruses has always been a fascinating topic, especially because of their unusual dual nature as a living and non-living entity. When a virus is in a live and receptive host, it reproduces its kind, but when it is in a non-living environment, it persists as an inanimate particle [2]. As a virulent pathogen, it exhibits a wide range of symptoms, rendering some viral infections mild and self-limiting, requiring only symptomatic care, whereas other forms, such as human immunodeficiency virus (HIV) infection, necessitate long-term medication [3]. The scientific community worldwide is working hard to develop a strategy to fight against these infectious viral diseases. In such a prevailing scenario, the design and delivery of a wide variety of innovative anti-SARS-CoV-2 therapeutics agents are desperately needed, not only to tackle COVID-19 disease but also to suppress a broad spectrum of previously resistant infectious virions and their mutants to save the human population from numerous life-threatening outbreaks. In addition, to successfully handle the COVID-19 pandemic it is important to develop safe and effective vaccines. From concept to clinical trial in humans, the main concepts and difficulties faced in the production of vaccines targeting a diverse range of pathogens need to be elucidated and tackled. In such a context a deep knowledge of the functioning of the immune system and host-pathogen interactions must be acquired for the efficient design of a novel vaccine candidate [4]. Vaccine production necessitates the identification of immunogenic epitopes followed by various phases of clinical trials to ensure the vaccine’s safety and efficacy profile.

Various SARS-CoV-2-based vaccines are in the final developmental stages and have been evaluated in a variety of in vivo models [5]. However, despite positive outcomes in animal models, only a handful of these vaccines have made it to humans. With access to several coronavirus genome sequences, bioinformatics methods can now be adopted to investigate the developmental origins of new strains as well as the mechanism of viral entry and pathogenesis in the host.

The present review focuses on the various stages of the vaccine developmental processes and available vaccines against COVID-19. It further explores the availability of popular tools and databases that are available for the identification and analysis of coronavirus, including molecular docking, vaccine and drug discovery, comparative genomics analysis, and other applications, for the better understanding of the pathogen at the genomic and molecular level. Therefore, the information and resources discussed in this review will certainly facilitate scientists in their ongoing research and development activities aimed at developing COVID-19 disease-fighting strategies.

## 2. Strategies for Vaccine Development

Immunization is a method for boosting the host immune system to develop immunological memory against a specific pathogen to confer protection from infection. Some vaccines are available that are composed of whole viruses or bacteria that can replicate [6]. They may contain microbial elements such as pathogen-associated molecular pattern molecules (PAMPs), which activate the innate immune system [7]. Nonetheless, in practice, a whole-pathogen strategy may not be effective or desirable from a safety standpoint, particularly if the agent is highly reactive or tumor-inducing. Recombinant DNA technology or chemical purification is another option that can be suitably employed to make a pathogen sub-unit as a vaccine antigen. These approaches necessitate in-depth comprehension of the pathogen’s biology. Adjuvants are utilized to increase and regulate immune responses by offering innate/PAMP triggers, thus accelerating a protective response to the pathogenic menace, since purified proteins have low immunogenicity on their own. In the formulation of any new vaccine, choosing the right antigens and adjuvants to optimize the downstream adaptive immune response is critical. From conception to clinical trial in humans, there is a need to explore the fundamental principles and obstacles encountered in the development of vaccines targeting a wide array of pathogens. The current pandemic of COVID-19 is a global epidemiological challenge; indeed, the true selection and development of the vaccine production platforms will require safe and effective methods as well as the time to produce millions of doses [8]. According to the last update information on 19 June 2021, the 322 vaccine candidates are in several phases of clinical trials (28 in Phase I; 30 in Phase I/II; 8 in Phase II; 24 in Phase III, and 8 in Phase IV), whereas 98 are in clinical testing.

### 2.1. Biology and Structure of the Pathogen

To classify antigens appropriate for the pathogen, it is crucial to have a comprehensive understanding of the pathogen’s biology and structure as well as its interactions with cellular receptors and the disease-causing processes for disease prevention [9]. It is crucial to understand the pathogen’s entry point and associated replication centers as the pathogens follow multiple routes to enter the biological environment, such as through injury, respiratory tract, genital tract, or gastrointestinal tract, and this therefore necessities differential vaccination strategies [10]. It is also pertinent to have a fair idea of the demographics of the prevalence of infection and a high-risk group of patients, as help to decide how and when to immunize [11]. It is important to provide specific criteria for a diagnosis, and these diagnostic methods should progressively include the ability to recognize both serotype and pathogen.

### 2.2. Initiation of Immune Response

The immune response that must be elicited by vaccination is the central objective in vaccine development. Since many pathogens entail receptor-mediated binding to cells and/or fusion, or initiate pathogenicity by generating particular toxins, antibody-mediated neutralization has historically been the primary target of a majority of vaccines [12]. More specific pathogen vaccines are structured to strengthen other facets of the innate and adaptive response. Antibodies, CD4^+^ and CD8^+^ T cells, and innate immunity all play a role in infection prevention and need to be tested and extended to vaccine-induced primary immunity or immunotherapy for chronic or recurrent infections [13]. It is also important to understand the function of specific T-cell-mediated effectors, such as antibody-mediated immunity support and pro-inflammatory cytokine production.

### 2.3. Vaccine Formulation and Manufacture

Vaccines must be manufactured effectively and administered in a manner that is suitable to the recipient. Whole species, either alive or dead, were used in the initially developed vaccines. There are certain advantages and disadvantages associated with the usage of either of the forms. The benefit of whole species is that they are extremely immunogenic, and they usually elicit a response close to that of natural infection [14]. However, they can cause pathology identical to that caused by natural infection, and a whole organism vaccine may not be effective in situations where natural infection does not produce protective immunity. The fact that whole organisms are intricate systems of carbohydrates, lipids, and proteins, which can greatly exacerbate production and quality control, is an added limitation. Choosing the best antigens that will ensure a powerful, sustained, and broad immune response of the kind required for defense while avoiding or minimizing reactogenicity is crucial. The novel technologies that are followed for vaccine design currently include purified proteins, protein–polysaccharide conjugates, viral/bacterial vaccines, recombinant DNA technology, and pathogen-like particles [15,16].

Other vehicles that facilitate the close integration of antigen and immunomodulators have been designed concurrently with the production of improved adjuvants for vaccine delivery. Toxoids, virosomes, liposomes, immune-stimulating complexes (ISCOMS), and micro- or nanoparticles are just a few examples [17].

Vaccines that require multiple doses involving the use of several diverse components or distinct vaccine technologies for priming and boosting can enhance outcomes in theory, but they are too complicated or costly to be practical [18]. The formulation is crucial in this situation. Hence, it is imperative on the part of researchers to judiciously select a formulation method that best suits the involved cost and guarantees superior therapeutic efficacy.

### 2.4. Pre-Clinical and Clinical Evaluation of Vaccines

Evaluating the potency or effectiveness of novel vaccine formulations continues to be one of the most complicated facets of vaccine design. The definitive proof of the effectiveness of the vaccine is necessary to ensure human safety; however, additional surrogate interventions are sought when selecting which vaccine candidates to undergo clinical trials [19]. Outlining the host–pathogen interplay, decoding the protective immune pathways implicated, and choosing the optimal adjuvant and antigen to accomplish the preferred immune response are all salient processes along the preclinical pipeline. The advancement of toxicological tests and immune readouts to determine the safety and performance of the candidate vaccine construct in pre-clinical assessment follows the development of the compliant vaccine delivery system. Although preclinical toxicology trials are typically smaller and often fail to detect direct toxic impacts, in vivo models have validated their significance for assessing vaccine safety and toxicology [20]. Human challenge investigations, in which volunteers agree to be revealed after vaccination, are the closest proximity models to human diseases in instances where an effectual therapy against the disease is obtainable. Until vaccine candidates are officially launched, they usually require a significant amount of research into their history, which enhances the safety profile and the desired therapeutic effectiveness.

## 3. Computation Approaches and Online Databases: Scope for the Pre-Validations

**Figure 1:**
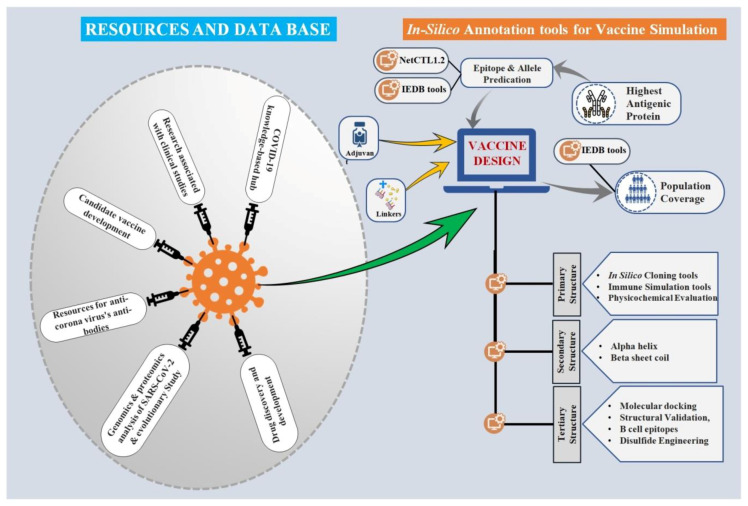
Computational approaches and online databases. The freely accessible integrated platforms can also be used as tools for coronavirus identification, analytical genomics evaluation, vaccine and drug discovery.

The theory of essential immune production through the development of required B-cell and T-cell-mediated immune responses underpins the rational design of prospective vaccine-mediated defense. Traditional vaccines fill the void, but at a cost in terms of time, money, and protection. As a result, developing a novel vaccine that is tailored to lay the foundations of immunity to combat the looming pandemic is of the highest concern [21]. The immunoinformatics-based strategy has laid the foundation for the identification of SARS-CoV-2 epitopes for the development of vaccine candidates, which can also be employed to identify important viral T- and B-cell epitopes. The numerous bioinformatics databases, software, and web servers that extensively maintain various genomic, epidemiological, and biological data related to coronaviruses may be of help in the design of potential vaccine candidates (Table 1). These freely accessible integrated platforms can also be used as platform tools for extracting valuable knowledge from vast and complex data sets spread across multiple databases. Hence, the following section entails common tools and databases for coronavirus identification, analytical genomics evaluation, drug and vaccine discovery, molecular docking, and various implications in coronavirus research (Figure 1).

### 3.1. Resources for Candidate Vaccine Development

#### 3.1.1. DBCOVP: Resources for Cross-Genome Comparison and Structural Virulent Glycoproteins

The Database of COronavirus Virulent glycoProteins (DBCOVP; covp.immt.res.in/ accessed on 21 July 2021) is a manually curated web server that is a complete repository of structural virulent glycoproteins, viz., nucleocapsid, membrane, envelope, and spike proteins from the 137 coronaviruses strain belonging to betacoronavirus genera [22]. At present, the DBCOVP harbors 185 protein sequences (47 spike proteins, 43 envelope proteins, 46 membrane proteins, 49 nucleocapsid proteins) from the human, bat, murine, bovine, rat, rabbit, equine, hedgehog species belonging to five subgenuses of the betacoronavirus, viz., *Embecovirus*, *Sarbecovirus*, *Merbecovirus*, *Hibecovirus*, and *Nobecovirus*. Each entry in the DBCOVP includes four significant annotation components, namely, Summary, Structural Details, Physicochemical properties, and Epitopes. The Summary section includes the strain name, associated organisms, taxonomic lineages, genomic location, subcellular localization, gene ontologies, functional domain, family categorization, protein and nucleotide sequences, and cross-referenced links to external databases like NCBI, KEGG, and UniProt. Likewise, the Structural Details section encompasses the secondary structural elements of all the protein entries, including the transmembrane helices; disulfide bond position; the number of helices, beta-sheet, and turns; the presence of signal peptides; predicted disorder region and cleavage sites; ubiquitination site details; the position of repeat sequences; and experimentally determined structures of the protein. Moreover, it enables the users to visualize and retrieve (in PDB format) the three-dimensional structure of the protein.

The third section contains a plethora of physicochemical properties for each protein, viz., the number of +ve and –ve charged amino acids, theoretical PI, instability index, GRAVY, aliphatic index, solubility, and hydropathy plot. The next section comprises immunogenic information on promiscuous epitopes, such as the binding Class I and Class II HLA alleles, toxicity, conservancy score, antigenicity, allergenicity, hydrophilicity, hydropathicity, molecular weight, charge, population coverage analysis of the predicted peptides. In addition, the three-dimensional predicted structures of the epitopes along with their docked complexes with binding HLA have been developed and can be directly accessed and downloaded. To further facilitate the data analysis, the DBCOVP provides built-in bioinformatics tools for phylogenetic tree construction, multiple sequence alignment, a compare tool (to perform comparative genomic analysis), and a local BLAST alignment search. This database also supports the submission of protein sequences in FASTA format, through the Data Submission Form, to further proceed with the comprehensive sequence–structure analysis.

#### 3.1.2. CoronaVIR: Multi-Omics Resource for Literature and Internet Resources

Computational Resources on Novel Coronavirus (CoronaVIR, webs.iiitd.edu.in/raghava/coronavir/ accessed on 21 July 2021) is a multi-omics resource that comprises valuable insights into the therapeutic, proteomic, genomic, and diagnostic information of SARS-CoV-2 coronaviruses, retrieved from the existing databases and literature [23]. All the information available on this holistic platform is categorized into five modules, viz., “General”, “Genomics”, “Diagnosis”, “Immunotherapy” and “Drug Designing”. The General module contains information on the updated WHO status, literature resources, current world trends, prevention guidelines, and links to external databases. The Genomics module harbors genome data belonging to various coronavirus strains to aid genomic level variations analysis. Concurrently, the Diagnosis module offers up-to-date insights on possible diagnostic tests to combat COVID-19, including five novel universal primer sets predicted by utilizing in silico approaches. This section supplies information on 12 experimentally validated and 65 unique predicted primer pairs. The Immunotherapy or vaccine design module comprises B-cell and T-cell epitopes that might evoke antibody-mediated immunity and cellular immune responses to counter infections caused by coronaviruses. It contains 1560 peptide sequences (of which 594 are B-cell epitopes and 966 are T-cell epitopes), 31,215 MHC class I binders, 17,635 MHC class II binders, and 117 experimentally validated IEDB assays linked to corona antigens. The assay information includes PubMed ID, epitope description, host organism, the technique used, assay group, and qualitative measure. Supported by the analysis of diverse prediction techniques, CoronaVIR suggests 17 epitopes as potential vaccine candidates against COVID-19. Subsequently, the drug module gives structural details of crucial FDA-approved drug molecules, repurposing drugs, monoclonal antibodies, and drug targets.

#### 3.1.3. CoVdb: Annotation Data Resources of Coronavirus Genes and Genomes

The Coronavirus database (CoVdb; http://covdb.popgenetics.net accessed on 21 July 2021) is an online genomic, proteomic, and evolutionary analysis platform that includes 5709 coronavirus strains belonging to various host species from approximately 60 countries [24]. CoVdb offers extensive information on gene function, subcellular localization, topology, and protein structure. The database includes in-built search tools to examine and study the evolutionary relationship between various documented coronavirus genomes utilizing the phylogenetic tree. CoVdb also confirmed pangolin as a potential intermediate host of SARS-CoV-2. It includes more than 300,000 GO records and approximately 50,000 function annotations. The CoVdb genome browser interface provides detailed information on various gene segments by utilizing Fst, Tajima’s D, CLR, and Pi analysis tracks. CoVdb includes a powerful search engine to browse taxonomy and supports sorting, filtering, BLAT, and BLAST. To facilitate the ongoing coronavirus research on tracing origination, vaccine, or drug design, CoVdb supports tools such as Protein, Aln Browser, Pop Analyzer, and Phylo Tree.

The Protein tool lists protein structural information and allows performing online protein structure analysis by an embedded application ‘iCn3D’. The Aln Browser tool permits easy retrieval of multiple sequence alignment of two or more strains at some position and constructs a phylogenetic tree using the alignment. The Phylo Tree tool allows visualizing and exploring phylogenetic trees constructed based on their genomic or proteomic sequences. Moreover, by using this database researchers can easily identify and retrieve complete genomic details based on coronavirus and can also perform comparative genomics, protein structure, and evolutionary analysis.

To unveil the origin of SARS-CoV-2 and further explore the recombination events between isolates from humans, bats, and pangolins, Zhu et al. explored the CoVdb repository and discovered that isolates belonging to bats had the highest number of subclades among reported hosts, indicating that coronaviruses in bats may have differentiated at higher levels than those from other hosts, owing to the population structure [25]. In the same study, CoVdb was utilized to identify and retrieve the coronavirus genomes, to further obtain an overall and general view of possible recombination events within the reported coronaviruses. Subsequently, annotation of the recombination events and population genetic analysis was also provided by CoVdb. In another study on the rapid wide spread of mutant alleles in SARS-CoV-2 strains, Zhu et al. employed CoVdb to identify, retrieve, and annotate consensus sequences of SARS-CoV-2 strains [26].

#### 3.1.4. hCoronavirusesDB: Comprehensive Resources for Genetic and Proteomic Data

The hCoronavirusesDB (http://hcoronaviruses.net/#/ accessed on 21 July 2021) is an integrated database and analysis resource covering the highly pathogenic human coronaviruses of SARS-CoV, MERS-CoV, and SARS-CoV-2. hCoronavirusesDB is funded by King Abdulaziz City for Science and Technology (KACST) and is supported by Imam Abdulrahman Bin Faisal University (IAU) and King Fahad University of Petroleum and Minerals (KFUPM). It houses a plethora of experimentally verified B-cell and T-cell epitopes for these pathogenic CoVs. Furthermore, it offers a BLAST tool to perform customized searches within the SARS-CoV, MERS-CoV, and SARS-CoV-2 database. Additionally, it also supports a standalone CLUSTAL-Omega sequence alignment tool and a geographic map distribution tool. The resource is useful for researchers looking into molecular tracking, vaccine development, and improved diagnostic tools for human coronaviruses.

### 3.2. Resources for Genomics Proteomics and Evolutionary Analysis of SARS-CoV-2

#### 3.2.1. SARS-CoV-2 3D: Comprehensive Resource for Proteome and Computational Modeling

SARS-CoV-2 3D (https://sars3d.com/ accessed on 21 July 2021) is an inclusive repository of the structures of gene products and their higher-order assemblies, i.e., homo- and hetero-oligomers and trans-membrane regions, including ligand and metal–ion interactions, with assessment scores [27]. The SARS-CoV-2 3D database provides monomers or homo- or hetero-oligomeric models for 21 proteins that are partially solved or do not have any experimentally determined 3D structures. The longest modeled protein is papain-like proteinase (PLpro) Nsp3, with 1945 residues, whereas the smallest is Nsp11 with 13 residues. This database also provides 308 structures of experimental viral–human protein–protein interactions to increase our understanding of how SARS-CoV-2 manipulates and disrupts cellular processes. Moreover, these viral–human protein–protein interactions are mapped to a 2D graph by employing D3.js Force-Directed Graph; a node in the graph represents the SARS-CoV-2 protein, while the edges represent the human proteins. Likewise, the dockings between small molecule ligands and target receptors such as Nsp3, Nsp5, Nsp12, Nsp14, Nsp15, Nsp16, and S protein are included in the database. These docked poses can be downloaded as PDB files, and the interatomic interactions formed by the ligands with the residue environment can be further visualized using the MolStar viewer (https://molstar.org/ accessed on 21 July 2021). Moreover, all the anti-viral FDA-approved drugs are screened and are mapped to DrugBank (www.drugbank.ca accessed on 21 July 2021) to provide initial information on the potential of repositioning of these drugs to act on specific target proteins in SARS-CoV-2. The SARS-CoV-2 3D database provides a user-friendly and easily accessible web interface to navigate, inspect and download the 3D structural proteome data, visualize modeled oligomeric complexes, analyze pockets of modeled structures, and investigate SARS-CoV-2 human–protein interactions, mutations, and protein–ligand docking.

In an in silico approach to track SARS-CoV-2 Nsp1 structural variants, Rezaei et al. exploited the SARS-CoV-2 proteome 3D database to identify and retrieve the complex structure of the Nsp1 with the ribosomal 40S subunit and associated interaction details [28].

#### 3.2.2. COVIEdb: Resources for Immune Epitopes of Coronaviruses

The database for potential immune epitopes of coronaviruses (COVIEdb; http://biopharm.zju.edu.cn/coviedb/ accessed on 21 July 2021) stores details on potential B- and T-cell epitopes for SARS-CoV, SARS-CoV-2, and MERS-CoV that are potential targets for pan-coronavirus vaccine development [29]. For easy and rapid retrieval of data, COVIEdb supports four main interfaces “B cell epitope”, “T cell epitope”, “Peptide”, and “Validated”. The B cell epitope interface maintains the details on B-epitopes, while the T-epitope interface supports the information on T cell epitopes, the Peptide interface contains the combined result of previously predicted B cell epitopes and T cell epitopes, and the Validated interface holds the predicted B and Tcell epitopes that have been corroborated by experimental studies. At present, there are 116 validated epitopes on the Validated page. Moreover, based on the predicted B cell epitopes and T cell epitopes, COVIEdb revealed 77 peptides that exist in all coronaviruses and have the potential to induce T-cell activation, and 10 of them have B_score greater than 4. The developed database not only can aid in vaccine development but also can suggest potent targets for drug designing.

#### 3.2.3. GISAID: Rapid Sharing of Data from Influenza Viruses and Visualization

The Global Initiative on Sharing All Influenza Data (GISAID; https://www.gisaid.org/ accessed on 21 July 2021) consortium mainly supports robust distribution of the available influenza virus’s information involving the recently detected, SARS-CoV-2 virus [30]. The database also harbors geographical site information in addition to species-specific data to assist in understanding the evolutionary relationships among the viruses and their distribution across geographical regions. In view of the significance of genetic information in enhancing our knowledge of the evolution of an infectious disease like COVID-19, GISAID encourages research collaboration among scientific communities based on open data sharing before publication. To promote efficient research, GISAID currently harbors various SARS-CoV-2-specific databases such as ViruSurf (http://geco.deib.polimi.it/virusurf_gisaid/ accessed on 21 July 2021), Mutation Situation Reports (https://outbreak.info/situation-reports accessed on 21 July 2021), CoVariants (https://covariants.org/ accessed on 21 July 2021), Covid-Miner (https://covid-miner.ifo.gov.it/app/home accessed on 21 July 2021), CoVizu (http://filogeneti.ca/covizu/ accessed on 21 July 2021), COVID-19 CoV Genetics Browser (https://covidcg.org/?tab=location accessed on 21 July 2021), NAAT Amplicons (https://covid-19-diagnostics.jrc.ec.europa.eu/amplicons accessed on 21 July 2021), Sequence Analysis Pipeline (https://cov.lanl.gov/content/index accessed on 21 July 2021), CoV-GLUE (http://cov-glue.cvr.gla.ac.uk/ accessed on 21 July 2021), Genomic Signature Analysis (https://covid19genomes.csiro.au/ accessed on 21 July 2021), Geographic Mutation Tracker (https://www.cbrc.kaust.edu.sa/covmt/ accessed on 21 July 2021 Global Testing and Genomic Variability (https://bioinfo.lau.edu.lb/gkhazen/covid19/genomics.html accessed on 21 July 2021), GESS (https://wan-bioinfo.shinyapps.io/GESS/ accessed on 21 July 2021), and Status of Detection Systems (http://penelope.unito.it/sars-cov-2_detection/ accessed on 21 July 2021). Various comparative genomic studies on the novel SARS-CoV-2 have used a GISAID database for retrieving both complete and partial nucleotide sequences. Recently, several comparative genomic studies on the novel SARS-CoV-2 used a GISAID database for retrieving both complete and partial nucleotide sequences [31,32,33].

#### 3.2.4. CoV3D: Structure-Based Design of Vaccines and Therapeutics against SARS-CoV-2

The database of high-resolution coronavirus protein structures (CoV3D, https://cov3d.ibbr.umd.edu/ accessed on 21 July 2021) is a user-friendly and constantly updated resource for coronavirus protein structures [34]. It presents comprehensive sets of 3D structures of coronavirus proteins and their interactive complexes with antibodies, receptors, and small molecules. Major components involved in the CoV3D database are interrelated tables, data sets, and tools for coronavirus protein structures and spike glycoprotein sequences. The structural part of CoV3D presents dedicated pages and tables for spike glycoprotein structures and conformational classification; antibody structures; spike structures with modeled glycans; main protease structures; protein structures belonging to nucleocapsids, NSPs, ORFs; major histocompatibility complex structures of coronavirus peptides. The CoV3D database can be a potent reference for the scientific community by presenting a user-friendly and up-to-date interface for coronavirus 3D-structural models, with integrated molecular viewers, structural classification, and a variety of other useful features. CoV3D facilitates the use of these growing structural data sets in analysis, modeling, and structure-based design efforts by collecting and annotating these structures and their conformations, and by providing inline structural viewing of single and multiple complexes.

#### 3.2.5. COVID-Profiler: Visualization of Multiple Genomic and Immunoinformatic Meta-Analyses

COVID-Profiler presents a plethora of tools that enable users to analyze SARS-CoV-2 sequencing and immunological data [35]. Primarily, COVID-Profiler provides three tools: (i) Profile, (ii) Phylogeny, and (iii) Diagnostics. The profile tool analyzes the whole genome sequence data and presents a report on the mutations detected. The phylogeny tool aligns multiple sequences and produces a maximum likelihood phylogeny tree. The diagnostics tools detect conservation of primer binding sites across the timeline of the pandemic in various geographic regions through interactive plots. COVID-Profiler also harbors significant online “immuno-analytics” tools that combine epitope, sequence, protein, and SARS-CoV-2 genetic variation analysis. This tool is available online from http://genomics.lshtm.ac.uk/immuno accessed on 21 July 2021. The source code for the web site and up-to-date raw data files are available at https://github.com/dan-ward-bio/COVID-immunoanalytics accessed on 21 July 2021. The SARS-CoV-2 immuno-analytics platform enables the visualization of multidimensional data to inform target selection in vaccine, diagnostic, and immunological research. The database underpinning the online tool is updated automatically using data parsing scripts that require minimal human curation. The monitoring of the temporal changes in the frequencies of mutations or their presence in multiple clades in a SARS-CoV-2 phylogenetic tree can provide insights into infection control, including post-vaccine introduction. Importantly, both the open access platform and the tool offer the acquisition of all the aforementioned data associated with the SARS-CoV-2 proteome, assisting further important research on COVID-19 control tools.

#### 3.2.6. VirHostNet 2.0: Analysis and Visualization of Virus/Host Protein–Protein Interactions Network

VirHostNet 2.0 is a knowledgebase resource specific to the network-based exploration of virus–host protein–protein interactions (http://virhostnet.prabi.fr accessed on 21 July 2021) [36]. In view of the current pandemic situation, VirHostNet 2.0 was upgraded (in March 2020) and now includes a comprehensive collection of protein–protein interactions manually annotated from the literature involving ORFeomes from multiple coronaviruses, including MERS-CoV, SARS-CoV-1, and SARS-CoV-2. The resource now includes a “SARS-CoV-2 release” that shows real-time, reproducible, and fair share systems biology research on COVID-19. This bio-curation effort also incorporated, in close to real time, the data obtained through affinity-purification mass spectrometry by the Korgan laboratory. VirHostNet 2.0 provides more than 650 binary protein–protein interactions that were made available to researchers working on COVID-19. The VirHostNet SARS-CoV-2 release will accelerate research on the molecular mechanisms underlying virus replication as well as COVID-19 pathogenesis and will provide a systems virology framework for prioritizing drug candidate repurposing.

### 3.3. Resources for Drug Discovery and Development

#### 3.3.1. CUReD: Web-Based Resources of Currently Available Drugs against SARS-CoV-2

CSIR Ushered Repurposed Drugs (CUReD; https://iiim.res.in/cured/ accessed on 21 July 2021) is a web server that provides information regarding the drugs, diagnostics, and devices, including the current stage of the SARS-CoV-2 trials (Council of Scientific and Industrial Research) of CSIR Partnered Clinical Trials. Developing novel drugs to treat COVID-19 infections and testing for their efficacy and safety will take several years. Thus, efforts to globally fast track and test the drugs approved or tested for other viral diseases such as HIV or Ebola, i.e., also called drug repurposing, can be a promising approach. Apart from carrying out its clinical trials, India is also participating in some of these ongoing global trials. For instance, CSIR is exploring all possible options, ranging from repurposed drugs to new drugs to AYUSH products and biological therapeutics, including vaccines.

#### 3.3.2. CORDITE: Database on Drug Interactions Based on Literature Aggregation with Web Interface

The Curated CORona Drug InTERactions Database for SARS-CoV-2 (CORDITE, https://cordite.mathematik.uni-marburg.de accessed on 21 July 2021) combines and represents information from various published articles as well as preprints about potential drugs, targets, and their interactions. It enables users to access, sort, and download relevant data to conduct meta-analyses, to design new clinical trials, or to conduct a curated literature search [37]. CORDITE automatically retrieves publications from PubMed (https://www.ncbi.nlm.nih.gov/pubmed/ accessed on 21 July 2021), bioRxiv (https://www.biorxiv.org accessed on 21 July 2021), chemRxiv (https://chemrxiv.org/engage/chemrxiv/public-dashboard accessed on 21 July 2021), and medRxiv (https://www.medrxiv.org/ accessed on 21 July 2021) that detail various computational, in vitro, or case studies on potential drugs for COVID-19. It enables the user to directly retrieve information related to publications, interactions, drugs, targets, and clinical trials. Moreover, the effectiveness and interactions of specific drugs with their respective protein are also shown as positive/negative scores, which can direct future meta-analyses studies. CORDITE presents an easy-to-use web interface that allows using a broad variety of resources for meta-analysis, design of new clinical studies, or simple literature searches, to guide the clinical world to an overview of existing studies and to accelerate the global search for new treatments.

#### 3.3.3. LSHTM VaC Tracker: Database on Up-To-Date Information on All the COVID-19 Vaccine Candidates

The LSHTM VaC tracker, launched in April 2020, aims to collate up-to-date information on manufacture projections, approval or licensure status, and vaccine storage requirements of all COVID-19 vaccine candidates. According to the information available in January 2021, the featured 291 COVID-19 vaccine candidates comprise 156 registered studies spanning 70 separate candidates, of which 20 are currently undergoing phase III efficacy testing [38].

### 3.4. Resources Associated with Clinical Studies

#### CoV-RDB: Online Relational Database for Candidate Anti-Coronavirus Compounds

The Coronavirus Antiviral Research Database (CoV-RDB; covdb.stanford.edu accessed on 21 July 2021) contains over 1800 cell cultures, 465 entry assays, 519 biochemical experiments, 259 animal model studies, and 71 clinical studies from over 400 published papers. SARS-CoV-2, SARS-CoV, and MERS-CoV account for 85% of the data [39]. The CoV-RDB includes four types of antiviral experimental information, viz., cell culture and entry assay experiments, biochemical experiments, animal model studies, and clinical studies; six main explanation tables to give details on viruses, virus strains/isolates, tested compounds, compound targets, cell types, and animal models; and a registry of ongoing or planned clinical trials. The Coronavirus Antiviral Research Database (CoV-RDB) is designed to promote uniform reporting of experimental results; to facilitate comparisons between different candidate antiviral compounds; and to help scientists, clinical investigators, public health officials, and funding agencies prioritize the most promising compounds and repurposed drugs for further development.

### 3.5. Resources for Anti-Coronavirus Anti-Bodies

#### CoV-AbDab: Resources for the Sequence–Structural Information and Metadata

The coronavirus antibody database (CoV-AbDab, http://opig.stats.ox.ac.uk/webapps/covabdab/ accessed on 21 July 2021) documents sequence–structural information and metadata on all pre-printed, published, and patented anti-coronavirus antibodies [40]. At present, CoV-AbDab contains nearly 1400 published or patented nanobodies and antibodies that are known to bind to at least one betacoronavirus. CoV-AbDab represents the first and only repository hub of antibodies known to bind betacoronaviruses, viz., SARS-CoV-2, SARS-CoV-1, and MERS-CoV. Moreover, it comprises important metadata involving evidence on cross-neutralization, antibody/nanobody origin, full variable domain sequence (where available) and germline assignments, epitope region, links to relevant PDB entries, homology models, and source literature. Researchers can use CoV-AbDab to yield new insights, including deriving crucial sequence/structural patterns that distinguish neutralizing from non-neutralizing SARS-CoV-2 binders [41], or deducing independent neutralizing epitopes exploitable by combination therapies [42].

### 3.6. COVID-19 Knowledge-Based Hub

#### COVID-19 Disease Map: Assembly of Molecular Interaction Diagrams

The COVID-19 Disease Map (https://covid.pages.uni.lu/ accessed on 21 July 2021) is a consortium of SARS-CoV-2 virus and host interaction mechanisms based on the input received from domain experts and guided by various published research [43]. This knowledge-based hub presents an efficient organization of the existing literature and the fast-growing number of novel publications on SARS-CoV-2, in both machine- and human-readable formats. This endeavor is an open collaboration among data scientists, life scientists, computational biologists, clinical researchers, and pathway curators. At present, 162 contributors from 25 countries are participating in the consortium, including partners from Reactome, WikiPathways, IMEx Consortium, Pathway Commons, DisGeNET, ELIXIR, and the Disease Maps Community. The COVID-19 Disease Map will be a promising resource for computational analyses and visual exploration of molecular processes involved in SARS-CoV-2 entry, replication, and host–pathogen interactions, including immune response, host cell recovery–repair mechanisms. Furthermore, the map will assist the scientific world and enhance our knowledge of this disease to support the advancement of competent therapies and diagnostics.

## 4. Vaccines for COVID-19: Efficacy and Prospective

**Figure 2:**
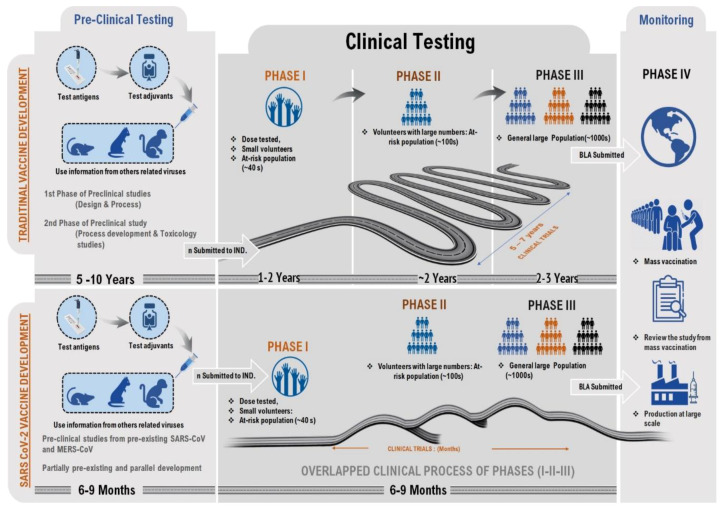
The comparative illustration of traditional vaccine production and CoV vaccine development. The reasons for focusing on each of these technologies for COVID-19 include their rapid expansion, scale, and manufacturing fit, and small dose size, which are all highly desirable features for a rapid pandemic response.

To avoid the substantial threat of a pandemic, investigators have been working around the clock to expedite the production and manufacture of a much-needed vaccine against COVID-19, and several of the emerging vaccine candidates have used the SARS-CoV-2 S protein as the main target [44]. Vaccine production necessitates the identification of an immunogenic epitope and the formulation of a complete vaccine after various trials have been completed to ensure the vaccine’s safety and efficacy profile. Although SARS-CoV-2 has a different genome organization, the research on SARS and MERS can help scientists to have a better understanding of the infection and immune response in the human body. Before initiating the mass vaccination stage and development process, the volunteer clinical trial is subjected to a code of conduct in terms of safety regulation of the vaccines. This clinical trial plan and approval for general use are strictly regulated by a governing body such as the Food and Drug Administration (FDA) and European Medicines Agency (EMA) [45]. The doses and schedule for the vaccine are calculated during restricted human studies and tested in phase III studies. Therefore, producing the vaccine against SARS-CoV-2 has the target time of only 12–18 months, while, historically, vaccines have taken 15–20 years to develop (Figure 2).

**Figure 3:**
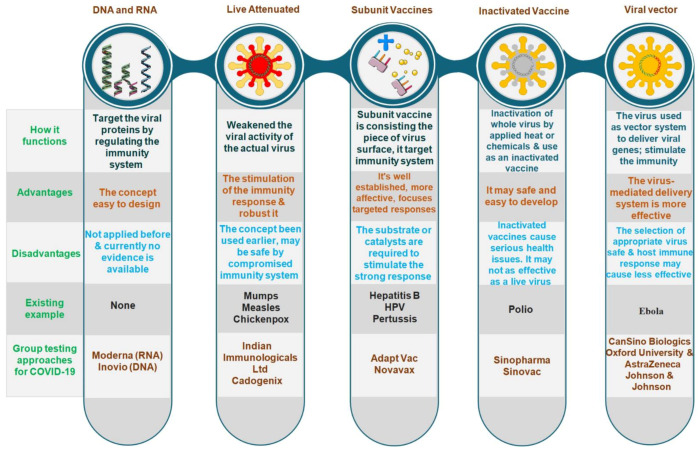
Types of vaccine development approaches and platforms. They differ in whether they use the entire virus or bacterium, only the germ parts that activate the immune system or only the genetic material that provides instructions for making specific proteins rather than the entire virus.

The COVID-19 vaccine candidates in development are focused on live attenuated viruses, DNA, RNA, nanoparticles, replicating and non-replicating viral vectors, immunogenic adjuvants, protein subunits, with each demonstrating its main advantages and risks before being released into the existing international market [46,47,48,49] (Figure 3). The new genetic method of the mRNA vaccine against SARS-CoV-2 is based on messenger ribonucleic acid (mRNA) fragments, the genetic material that is copied from DNA and encodes proteins. It can stimulate the production of antibodies, which can neutralize the virus in laboratory samples. Another type of DNA vaccine is obtained by recombinant DNA technology encoding with the target molecule. Modified ChAdOx1, nCoV-19, and INO-4800 are some of the new DNA vaccines against coronavirus that are entering human phase I testing.

The subunit vaccines concept is based on certain antigenic determinants and increases the efficiency of the immune response against pathogenic microorganisms. In addition, the development of a vaccine based on the surface proteins that form virus-like particles (VLPs) is a more innovative approach. The vaccine candidates that are already commercialized are presented in Table 2. Vaccine developers’ products such as Moderna’s mRNA1273 and Pfizer’s BNT162b2 (mRNA-based), Sino Biotech’s CoronaVac, Sinovac’s SARS-CoV-2 vaccine (inactivated virus), Johnson & Johnson’s JNJ-78436735, ChAdOx1 nCoV-19, Sputnik V (adenovirus-based), CanSino’s Ad5-nCoV (viral vector), Inovio’s INO4800 (DNA plasmid vaccine), considered to be the front-runners, are currently gearing up to conduct phase trials and manufacture up to a billion doses.

## 5. Conclusions

The development of a vaccine is a multidisciplinary endeavor that combines molecular knowledge of host–pathogen interactions, selection of antigens to deliver an immune response, formulation aspects, and pre-clinical and clinical testing of the developed vaccine to assure an optimum therapeutic efficacy and safety to human. An in-depth understanding of the immune processes underlying the disease and defense, and how they differ among individuals, risk groups, and populations is needed. The gained expertise helps in choosing the antigenic targets, as well as using the adjuvants and delivery systems to shape the immune response elicited by the vaccine, which, in turn, influences the manufacturing requirements and clinical trial architecture. Understanding the molecular and evolutionary origins of SARS-CoV-2, the underlying mechanisms of viral–host binding interaction, and the detection of potential antiviral peptides and epitopic vaccine candidates as potential therapeutic choices against coronaviruses has improved our knowledge of the coronavirus pathogenesis. These advancements have been supplemented by the emergence of novel computational databases and tools specifically for coronavirus research, which have not only strengthened research strategies to prevent COVID-19 but also aimed to consolidate the massive volume of genomic data and relevant research observations on freely accessible centralized platforms to disseminate to the wider scientific community worldwide.

## Figures and Tables

**Table 1:** Software/tools used for the development of vaccines against SARS-CoV-2 coronaviruses.

Categories	Resources	Web Link & Accessed Details	Utility
Resources for Candidate vaccine development	DBCOVP	http://covp.immt.res.in/ accessed on 21 July 2021	This web resource provides comprehensive knowledge on the complete repertoire of structural virulent glycoproteins, viz., spike, envelope, membrane, and nucleocapsid protein from the 137 coronaviruses strain belonging to βcoronavirus genera.
	CoronaVIR	https://webs.iiitd.edu.in/raghava/coronavir/ accessed on 21 July 2021	This multi-omics resource includes valuable insights into the genomic, proteomic, therapeutic, and diagnostic knowledge of novel SARS-CoV-2 coronaviruses curated from the literature and existing databases.
	COVIEdb	http://biopharm.zju.edu.cn/coviedb/ accessed on 21 July 2021	This database provides details on potential B/T-cell epitopes for SARS-CoV, SARS-CoV-2, and MERS-CoV to provide potential targets for pan-coronaviruses vaccine development.
	hCoronavirusesDB	http://hcoronaviruses.net/#/ accessed on 21 July 2021	An integrated database and analysis resource covering the highly pathogenic human coronaviruses of SARS-CoV, MERS-CoV, and SARS-CoV-2.
Resources focused on genomics proteomics and evolutionary analysis of SARS-CoV-2	SARS-CoV-2 3D database	https://sars3d.com/ accessed on 21 July 2021	A comprehensive database of the structures of all gene products and their higher-order assemblies, i.e., homo- and hetero-oligomers, and trans-membrane regions, as well as ligand and metal–ion interactions, with an acceptable assessment score.
	CoVdb	http://covdb.popgenetics.net accessed on 21 July 2021	An online genomic, proteomic, and evolutionary analysis platform that includes 5709 coronavirus strains (From 1941 to 2020) belonging to various host species from approximately 60 countries.
	GISAID	https://www.gisaid.org/ accessed on 21 July 2021	This consortium mainly supports rapid sharing of all influenza virus data, including the recently detected SARS-CoV-2 coronavirus.
	CoV3D	https://cov3d.ibbr.umd.edu/ accessed on 21 July 2021	This database presents comprehensive sets of 3D structures of coronavirus proteins and their interactive complexes with antibodies, receptors, and small molecules.
	COVID-Profiler	http://genomics.lshtm.ac.uk/ accessed on 21 July 2021	This web server includes a plethora of tools that enable users to analyze SARS-CoV-2 sequencing and immunological data.
	VirHostNet 2.0	http://virhostnet.prabi.fr accessed on 21 July 2021	A knowledgebase resource specific to the network-based exploration of virus–host protein–protein interactions.
Resources for drug discovery and development	CUReD	https://iiim.res.in/cured/ accessed on 21 July 2021	A web server that provides information regarding the drugs, diagnostics, and devices including the current stage of the trials on COVID-19.
	CORDITE	https://cordite.mathematik.uni-marburg.de accessed on 21 July 2021	This database combines and represents information from various published articles as well as preprints about potential drugs, targets, and their interactions. It enables users to access, sort, and download relevant data to conduct meta-analyses, to design new clinical trials, or to conduct a curated literature search.
	LSHTM VaC tracker	https://vac-lshtm.shinyapps.io/ncov_vaccine_landscape/ accessed on 21 July 2021	This database hosted by the Vaccine Centre (VaC) at the School of Hygiene and Tropical Medicine provides a user-friendly up-to-date view of the global vaccine landscape.
Resources associated with clinical studies	CoV-RDB	https://covdb.stanford.edu accessed on 21 July 2021	This database includes details of over 1800 cell cultures, 465 entry assays, 519 biochemical experiments, 259 animal model studies, and 71 clinical studies from over 400 published papers. SARS-CoV-2, SARS-CoV, and MERS-CoV account for 85% of the data.
Resources for anti-coronavirus anti-bodies	CoV-AbDab	http://opig.stats.ox.ac.uk/webapps/covabdab/ accessed on 21 July 2021	This portal presents sequence–structural information and metadata on all pre-printed, published, and patented anti-coronavirus antibodies.
COVID-19 knowledge-based hub	COVID-19 disease map	https://covid.pages.uni.lu/ accessed on 21 July 2021	A consortium of SARS-CoV-2 virus–host interaction mechanisms based on the input from domain experts and guided by various published works.

**Table 2:** List of approved/authorized vaccines.

Name of the Vaccine Candidate	Name of Developers	Platform Utilized	Country of Origin
Moderna COVID-19 Vaccine (mRNA-1273)	Moderna, BARDA, NIAID	mRNA-based vaccine	USA
Covaxin	Bharat Biotech, ICMR	Inactivated vaccine	India
Comirnaty (BNT162b2)	Pfizer, BioNTech; Fosun Pharma	mRNA-based vaccine	Multinational
Covishield	Oxford University and AstraZeneca	Adenovirus vaccine	UK
Sputnik V	Gamaleya Research Institute, Acellena Contract Drug Research, and Development	Recombinant adenovirus vaccine (rAd26 and rAd5)	Russia
CoronaVac	Sinovac	Inactivated vaccine (formalin with alum adjuvant)	China
COVID-19 Vaccine Janssen (JNJ-78436735; Ad26.COV2.S)	Janssen Vaccines (Johnson & Johnson)	Non-replicating viral vector	The Netherlands, USA
EpiVacCorona	Federal Budgetary Research Institution State Research Center of Virology and Biotechnology	Peptide vaccine	Russia
BBIBP-CorV	Beijing Institute of Biological Products; China National Pharmaceutical Group (Sinopharm)	Inactivated vaccine	China

## Data Availability

Data contained within the article are available in a publicly accessible repository/web link.
